# Use of Biomass-Derived Materials for Their Potential Addition to Car Bumpers: A Critical Review

**DOI:** 10.3390/polym17172402

**Published:** 2025-09-03

**Authors:** Cristiano Fragassa, Sofia Migani, Muhammad Awais, Orion Jucja, Zeeshan Mujtaba, Carlo Santulli

**Affiliations:** 1 Geology Section, School of Science and Technology, Università di Camerino, Via Gentile III da Varano 7, 62032 Camerino, Italy; cristiano.fragassa@unibo.it; 2 Department of Industrial Engineering, Alma Mater Studiorum Università di Bologna, Viale del Risorgimento 2, 40136 Bologna, Italy; 3 Chemistry Division, School of Science and Technology, University of Camerino, Via Madonna delle Carceri (ChIP), 62032 Camerino, Italy; sofia.migani@unicam.it (S.M.); muhammad.awais@unicam.it (M.A.); zeeshan.mujtaba@unicam.it (Z.M.); 4 National Center for Drug Research and Evaluation, Italian National Institute of Health, Viale Regina Elena, 299, 00161 Rome, Italy; 5 School of Pharmacy, University of Camerino, Via Madonna delle Carceri (ChIP), 62032 Camerino, Italy; orion.jucja@unicam.it; 6 School of Pharmacy, Virginia Commonwealth University, Richmond, VA 23298, USA

**Keywords:** car bumpers, lignocellulosic fibers, biopolymers

## Abstract

The large availability of biomass, which, for the sake of decarbonization, is not supposed to be disposed of by burning and energy recovery, has led to the increasing production of polymer composites containing biomass-derived materials. The automotive industry is a field which, due to large production volumes and the requirements for safety, has large economic and environmental value. Despite this, the introduction of lignocellulosic fiber composites and generally biopolymers into this sector has been slow and so far mostly limited to non-structural or semi-structural components. This review critically considers the difficulties associated with the production of bumpers with biomass-derived materials and reports on a variety of fibers and polymers that have been proposed and on the equally variable degree of success of these studies. We also report on the understanding that rethinking the bumper in terms of materials could be an effective way to introduce biomass-based materials into the whole automotive sector on a larger scale with increased benefits.

## 1. Introduction

One of the routes that could be explored to reduce the environmental impact of the whole fabrication process for automobiles is the introduction of biomass, including especially lignocellulosic fibers in structured format (e.g., textiles or mats), to accomplish especially important functional tasks, such as the protection of passengers [1]. Natural fibers have been considered for the production of motor cars for a long time. A historical trigger for their introduction was the design of Ford’s “hemp car”, which, for the first time, led to the identification of which parts of a car could be fabricated using this fiber, in a context that also included fueling vehicles using hemp bio-oil [2]. This concept has gradually become more manageable and practical, especially in view of the application of polymer resins in car fabrication, the first example of which was phenolic resin (Bakelite), plasticized with formaldehyde, which, as a matter of fact, was used for the iconic East Germany city car, the Trabant [3]. In this specific case, a number of parts, including the doors, roof, boot lid, bonnet, and fenders, were manufactured using a patented waste cotton-reinforced phenolic resin (Duroplast).

Over time, these two approaches have proceeded side by side in the following manner: (i) developing semi-finished products, including natural fibers, such as fabrics and pre-pregs, specifically for use in composites and (ii) using by-products or waste from other sectors, especially the textile industry, which is then adapted for use in composites, as will also be illustrated later on.

Recently, the availability of a large amount of biomass has made the latter approach more interesting and fashionable, though in practical terms, the amount of biomass that can feasibly be introduced into the car structure is normally lower [4]. Whilst initially the search for waste, lately defined as “secondary raw materials”, concerned industries with widely spread products in particular, such as wool and silk, as well as pineapple, hemp, sugarcane, and palm [5], later studies also considered niche fibers, which might present a localized but constant-over-time interest, if not even growing interest [6].

This review aims to address an important research gap, which is related to the fact that bumpers, as an essential part of the automobile structure, can be manufactured using biomass-filled materials, such as lignocellulosic fiber composites. This would also provide a non-negligible contribution towards the increased decarbonization of the automotive industry. The existing literature, which will be progressively discussed in this work, reports sparse attempts to propose the use of various biomasses for bumper production. However, a structured exploration of the principal projects with this objective is not available so far, and the advantages and difficulties encountered in the more general case of automobile structural components have not been extensively reviewed.

The search for bibliographic research, starting from already known sources, which have been collected by the corresponding author and their collaborators over the last few years, was centered on the use of Google Scholar, with more limited use of Scopus and Web of Science only in the case of conflicting or lacking information, between the years 2000 and 2025. This was carried out with the understanding that earlier works might be referenced as well to clarify the history and evolution of the process. This approach is particularly required for understanding the origins of materials, such as the use of natural fibers in materials, which is still proposed, though sometimes in an actualized way, for improved control over process parameters, alongside traditional solutions, e.g., alkali treatment of the fiber surface. The evaluation of the quality of the studies does not exclusively refer to the impact factor of the relevant journals or the number of citations over time for a specific paper. These metrics might be confusing, especially when considering niche fibers for this purpose, which have been researched in specific journals of local interest but are perfectly relevant for this study. Research has mainly concentrated on papers from journals possessing an impact factor at the time of their publication, with some attention paid to book chapters and very limited consideration of conference papers, only if the subject treated (e.g., the fiber examined) is completely unheard of in this application context. Patents were not considered, since the objective of this review is mainly focused on academic research, which offers prospective solutions and is of interest for future developments in this field.

**Figure 1:**
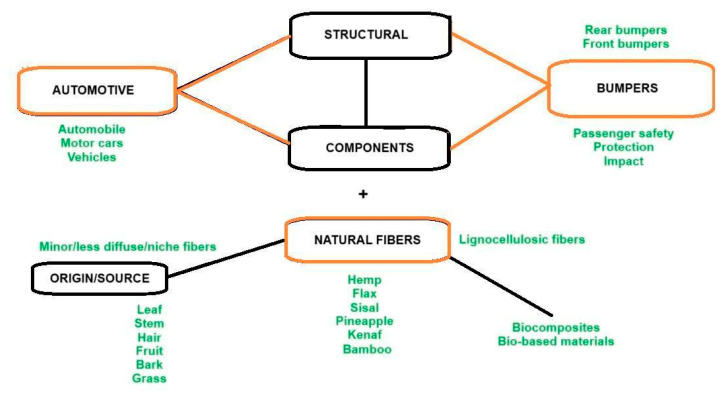
Scheme followed for the bibliographic research; the red-bordered words represent the base of the research, the black-bordered words are the additional/completion words, and the green words are the alternative words.

**Figure 2:**
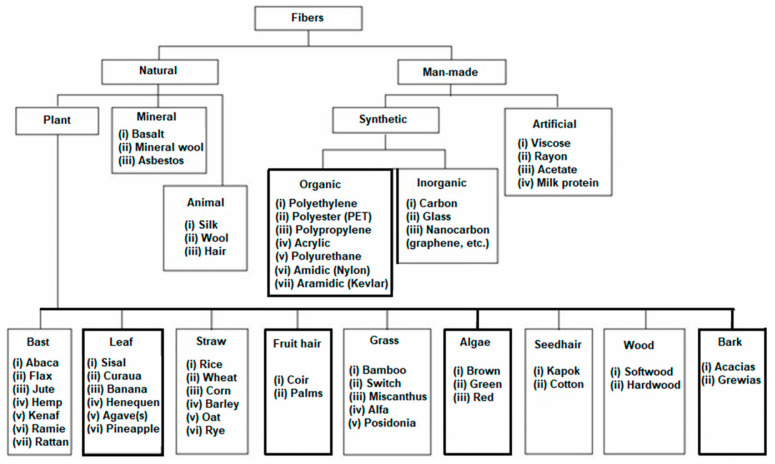
Classification of fibers for possible/actual use in composites [7].

A scheme of the keyword search is shown in Figure 1. Typically, the research methodology is carried out by using the combination of the three keywords “automotive”, “bumpers”, and “natural fibers”, circled in brown in the figure, together with other keywords from the upper part (structure) of the figure or its lower part (materials). For the identification of minor fibers used in the sector, the lowest line of the scheme reported in Figure 2 was also employed to amplify the search for natural fibers [7]. Regarding the content of composites, alternative definitions, such as “plant fiber composites” or “biomass composites”, were excluded from the scheme since they are less common and might result in excessive searching. However, it has been suggested that in the long run “biomass composites” might become a common term, since it appears that niche fibers are described as “biomass”. Biomass also includes woody materials though, which for the specific application on bumpers do not appear very relevant so far.

The structure of this work is analyzed in Section 2, outlining the gradual transition to a more bio-based automobile and the prospective role of car bumpers in it. More generally, in Section 3 typical non-metal materials for car bumpers are analyzed, such as glass fiber/polypropylene composites, offering indications of their performance, whilst in Section 4, general performance indications are reported for inherently biodegradable polymers, often defined as “bioplastics”, which are also slowly entering the picture. Section 5 clarifies the current role of bioplastics and natural fiber composites in the automotive industry, in the specific case of bumpers, therefore concentrating on the impact performance and crashworthiness of these materials. Finally, Section 6 discusses future perspectives on how biomass introduction will change the fabrication of a bumper, particularly as far as the use of biodegradable polymer composites is concerned.

## 2. Car Bumpers in an Increasingly Bio-Based Automobile

Originally, car bumpers were applied, rather than precisely designed, in order to prevent contact between an obstacle and the car body or to cushion the collision shock between vehicles; as a consequence, at the beginning of automobile development, simple beam-like metal structures were manufactured [8]. More recently, despite retaining a relatively simple geometry, car bumpers have more precisely and measurably been required to accomplish the physical task of absorbing the kinetic energy in an accident, first through elastically deflecting, then through plastically deforming and being damaged [9]. This process needs to happen to minimize harm both to pedestrians and other road occupants, such as cyclists, and car passengers [10]. In particular, according to European regulations for front and rear bumpers with certification ECE R-42, the front bumper system needs to be designed to protect the vehicle in the event of a low-speed (less than 8 km/h) crash, without suffering any visible damage at this velocity [11]. This implies that no damage to functionally relevant parts would be caused by this type of accident, and that, in the worst case scenario, only minimal plastic deformation of any other component of the vehicle would occur. On the other hand, ECE R-42 regulations do not provide a reference point from which deformation needs to be measured and therefore does not provide much information regarding the relationship between deformation and thickness. This was attempted in [12] for carbon fiber composites, indicating that point deformation may decrease to an extent much exceeding the reduction in thickness between bumper models, hence also affecting the collision end time. Adding biomass to the bumper would normally result in an increase in thickness and in complex mode impact absorption due to diffuse breakage across the bumpers, as reported by a study on impact hysteresis cycles in [13], where the stacking sequence played a significant role. Another factor of large importance for impact absorption is fiber length, which was investigated in detail for the application of palm frond fibers to bumpers in [14], indicating that increasing the length of fibers from 40 to 60 mm increased toughness by a proportional amount, and the tensile strength almost doubled. These factors would meet requirements in view of the lower cost of biomass [15], especially during a transitional period for the automotive industry such as the present one.

Beyond safety regulations, discussions on car bumper design and materials also received a considerable boost after the design on end-of-life (ELV) vehicles was modified and made more restrictive, with the aim of increasing the recyclability and bio-based content of automobiles [16]. In general terms, the amount of plastic that can be replaced in cars by bio-based materials is often less than 10% of the total weight of the structure, which leads to eco-labeling to demonstrate the extent and environmental added value of this substitution [17]. The diffusion of bioplastics and natural fiber composites has led to their gradually increased involvement in the automotive industry, which could assist in the development of purposely designed bio-based materials to extend their use to different parts of the car structure [18]. However, a considerable difference exists between components that are only used to complete cars and provide comfort, aesthetic value, and recognizability to the model and others, such as bumpers, that are essential for preserving automobile safety during operation. It could be considered that the fabrication of bumpers may act as a trigger to allow bio-content to be applied in an automobile through a really useful strategy and not just for purely “cosmetic” reasons or finishing. Through this statement, we do not intend to dismiss the difficulties of this operation. A long time has passed since the aforementioned “hemp car” was proposed by Henry Ford, which included a lignin-based binder for hemp (and partially soy) fibers for the fabrication of the car’s body, yet interestingly, its bumpers were still designed in a geometry that could be adapted for a classical steel structure [19]. This process generated considerable attention, namely on the introduction of lignocellulosic fibers into automotive composites, as mentioned in various reviews available on this topic (e.g., [20,21,22,23,24,25]). Some important outcomes in this field provided in the literature are reported in Table 1. Other reviews on specific lignocellulosic fibers in automotive applications also exist, which will be dealt with later on in this discussion. More recently, the potential of biodegradable matrix composites, such as poly(lactic acid) (PLA), in combination with lignocellulosic fibers, for car crashworthiness was also investigated to substitute traditional polymer composites in the external “shell” of a vehicle [26,27]. An intermediate approach is often selected, which involves the application of a hybrid composite, including both natural fibers and glass/carbon fibers, for the production of car bumpers [28].

## 3. Typical Non-Metal Materials for Car Bumpers and Their Performance

**Figure 3:**
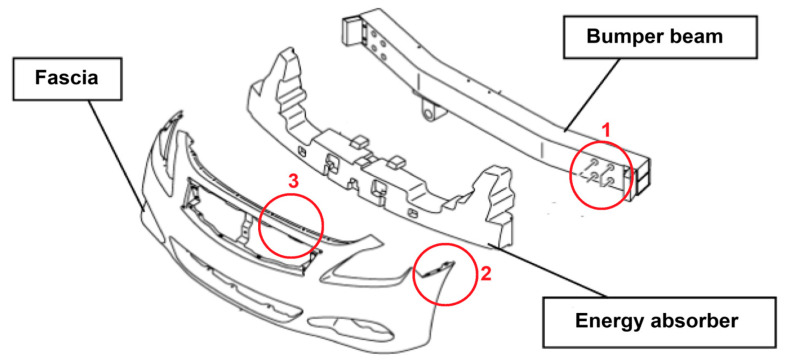
Scheme of a typical bumper with possible problems generated by the insertion of biomass into the structure: in particular, (1) the need for resistance around the holes for screwing; (2) difficulty of obtaining clean edges in the geometry; and (3) the requirement for constant thickness, especially in the thinner parts of the structures (redrawn from [32]).

**Figure 4:**
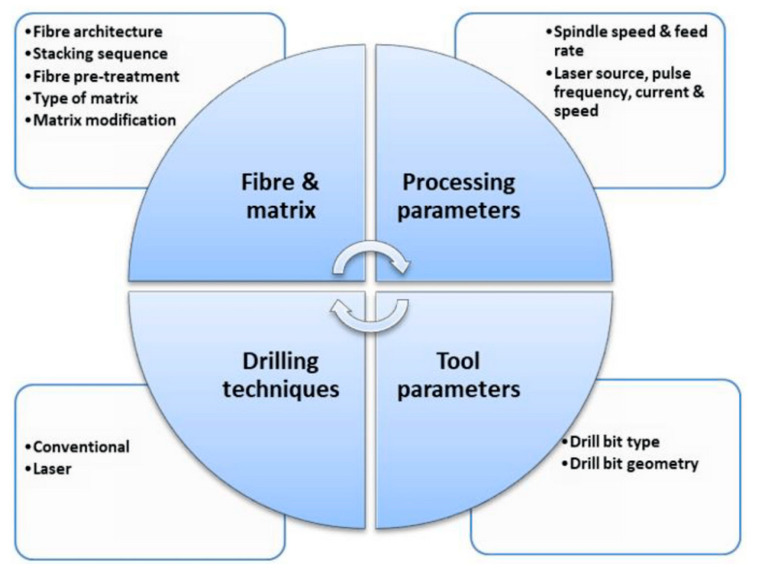
Factors involved in drilling and tightening operations in natural fiber biocomposites [34].

Most well-performing non-metal materials for car bumpers are composites based on carbon or nylon fibers, though the most obvious choice is still fiberglass with a polypropylene matrix or loaded polypropylene [29]. In the specific case of glass–PP composites, the improvement of the fabrication procedure and reduction in microstructural voids through methods such as combining polypropylene fibers with glass ones provided improved mechanical and impact properties [30]. Also, in recent applications on car bumpers, the importance of glass fiber-reinforced composites has been confirmed for their more progressive damage evolution during the impact event due to their hybrid nature [31]. The introduction of biomass in bumper structures, though it might contribute to impact damage absorption, does create a number of practical issues, which are reported in an expanded form in Figure 3, modified from a general study on bumper criticalities [32]. The least obvious challenge is that related to controlling hole dimensions and geometrical tolerance and damage during screw tightening in composites including biomass, which is dealt with in [33]. In this case, a combination of drilling and manufacturing parameters is examined to ensure a higher “drilling friendliness” of the biomass composite. Post-drilling properties are normally analyzed in a simpler way using tensile residual performance: this only limitedly and partially describes the difficulties encountered during operation: fibrillation is, for example, a common issue during tightening operations. In general, the result obtained highly depends on a number of factors, which are divided into various lists in Figure 4 [34].

This standardized product, based on glass fibers and the polypropylene matrix with mineral fillers, would also fulfil the ambitious objective of applying mechanical recycling to create a bumper-to-bumper process, which could be the best option for an operational and effective end-of-life vehicle (ELV) strategy. In principle, polypropylene (PP), as the principal matrix used in composite car bumpers, does offer some promise in terms of recyclability, being a thermoplastic with relatively facile reprocessability: in the practical case of automotive composites, mechanical recycling needs to account for the high content of glass fibers together with other ceramic fillers and the requirement for paint removal [35].

Whenever polypropylene matrix bumpers are proposed for recycling, we need to consider whether they should be loaded with ceramic fillers, usually talc [36], to improve hardness and wear resistance, together with rubber, typically an ethylene–propylene diene monomer (EPDM) and polyolefin elastomer (POE), to enhance impact strength and elongation, even at the expense of stiffness [37]. Talc has been recognized as an important ceramic particle filler in automotive PP for its higher stiffness (4.2 vs. 2.4 GPa) with respect to both calcium carbonate and barium sulfate [38]. Also, it is possible to incorporate talc into the production of cellulose-containing composites, such as injection-molded cellulose nanofiber (CNF)–PP composites, offering a linearly growing contribution to the enhancement of mechanical properties [39].

However, in view of a more sustainable industrial approach involving cost reduction and reduced resource depletion, more recently, ceramics, as secondary raw materials from other production systems, such as 95% pure calcium carbonate (calcite) from eggshells, have been proposed [40]. This in itself does not hinder injection molding of the composite, even using alternative matrices, such as poly(lactic acid) (PLA) [41]. The use of lignocellulosic fibers with eggshell powder in composites has also increased recently, including jute mat [42], jute/banana [43], and sisal fibers in hybridization with glass [44].

From the above limitations, it is no surprise that transitioning to practical recycling will by no means be smooth and free from quality degradation. In particular, a study indicated that the impact resistance of a recycled bumper made from neat polypropylene loaded with rubber and mineral fillers significantly decreased [45], remaining at values much lower than the suggested value of 35 kJ/m^2^ [46]. The use of recycled PP to produce a car bumper, using three configurations with a 70/10, 60/20, and 50/30 ratio between PP and rubber and a fixed 10% calcium carbonate and EPDM content, led to rather inconsistent results, never matching the performance level of new PP: the most critical factor was, other than impact strength, a loss of 20% in tensile strength [47]. The successful performance of polypropylene is linked to different factors; in particular, its large elongation facilitates energy absorption while minimizing fracturing. Other plastics able to offer sufficient rubbery and damage-tolerant behavior were also considered as bumper materials, such as acrylonitrile–butadiene–styrene (ABS) [48], which provides greater stiffness but slightly compromised toughness. In terms of crash behavior, it was reported that the ABS bumper was not as effective as other composite solutions, such as polyamide/30% glass fibers or epoxy/glass, despite being considerably lighter (ABS has a density of approximately 1.05 g/cm^3^) [49]. Common automotive plastics exhibit tensile strengths ranging from tens of MPa, such as approximately 30–50 MPa for polypropylene and 40–60 MPa for acrylonitrile butadiene styrene (ABS), alongside elongations varying from less than 10% for ABS to more than 100% for PP, illustrating these trade-offs [50].

On the other hand, a polyamide/30% glass fiber composite is by no means comparable with any composite solution including carbon fibers [51]. Despite these recognized limitations, the ease of production and limited costs, together with their low weight (just over 0.9 g/cm^3^ density), has sustained the use of PP or general thermoplastic polyolefins (TPO) as a standard polymer for car bumpers over time [52], while other possibilities are currently being investigated. Among these are polycarbonate (PC)/ABS blends, which offer additional rigidity or heat resistance compared to PP [53]: proposals have also been published on the use of polyesters, such as polyethylene terephthalate (PET) and polybutylene terephthalate (PBT) [54], and possibilities for blending and compatibilization are always wide open, which, even in the most debatable case, can offer improved processability [55].

To summarize, the recyclability of conventional bumper materials in thermoplastics is favorable in theory: PP, PET, and ABS are thermoplastics that can be re-melted and remolded. In practice though, merely a small portion of plastics from end-of-life vehicles are actually recovered, though obviously the use of thermoplastics would be most promising in this sense [56]. However, the presence of rubber and foam reinforcements, along with paint residues, poses significant challenges to the recycling process. Moreover, the environmental impact raises concerns: plastics derived from fossil fuels deplete resources during pre-production while releasing carbon dioxide during production.

## 4. Bio-Based and Inherently Biodegradable Polymers

To further reduce the environmental impact of automotive thermoplastic polymers, used as such or as the matrix for composites, and compensate for their difficult recyclability, some possibilities appear to be available:

Using bio-based polymers obtained from renewable sources, such as sugarcane, which implies reduced resource depletion: in particular, bio-PE has been proposed for use in the automotive industry [57], alone or in the form of a composite with curauà fibers [58].

Using inherently biodegradable polymers, which are assumed to be adequately protected during the service life of the material (e.g., through the application of paints and surface treatments) but can be decomposed more easily at the vehicle’s end of life by biological processes. The initial fields of application for these polymers were agriculture (controlled biodegradation), medicine (drug delivery), and packaging [59]. These are all sectors in which the material duration and structural properties required are quite limited, whereas the measurable biodegradation effect in some conditions (operation or post-operation) is much more important. Using inherently biodegradable polymers in the automotive industry would instead require extended durability for as long as possible, compatible with the life of the vehicle. For this reason, the effective protection of the material, e.g., through the application of paint layers, during the operational lifetime is of paramount importance. In this regard and in view of a lifecycle analysis (LCA) with improved outcomes, paints made from secondary raw materials, such as castor oil, are more suitable for this purpose [60].

This strategy (maintenance through the application of durable paints) leads to less energy consumption with respect to thermal recycling, especially regarding the limited energy involved, as it is usually carried out at ambient temperature [61]. Moreover, in terms of LCA, the thermal recycling option is not always very popular for traditional plastic bumpers, especially because of the larger costs involved, which appear to be scarcely compensated for, even by the governmental incentives provided, e.g., in the case of Japan [62]. More general criticisms were raised on the fabrication of plastic bumpers (in particular, unsaturated polyester ones from a manufacturer in Algeria) as compared with steel competitors due to the impact on workers’ health during the production process [63]. These examples suggest that in terms of environmental and even social sustainability, the application of plastic bumpers is not always a winning option. In this context, the introduction of biomass into a polymer matrix bumper may generate a number of options for recycling other than mechanical/thermal recycling and chemical recycling, which are available for all thermoplastics. In particular, other than composting in specific plants, another possibility is anaerobic digestion, leading to methane and further biomass [64]. In the case of composites, the inherent biodegradability of the matrix could enable the reprocessing of non-biodegradable fibers, e.g., the recovery of short fibers from fiberglass. This has been carried out with glass/polypropylene bumpers through melt processing [65]. The technical difficulty lies in allowing for matrix decomposition in the composting system while recovering glass fibers after mechanical grinding; thus, at the present time, biological recycling only appears possible when lignocellulosic fibers are used as the reinforcement for the composite, which will be the specific subject of Section 5.

**Figure 5:**
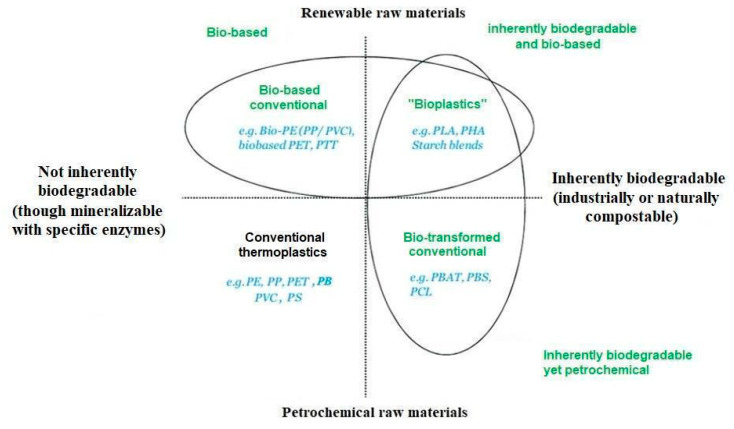
Classification of thermoplastics.

**Figure 6:**
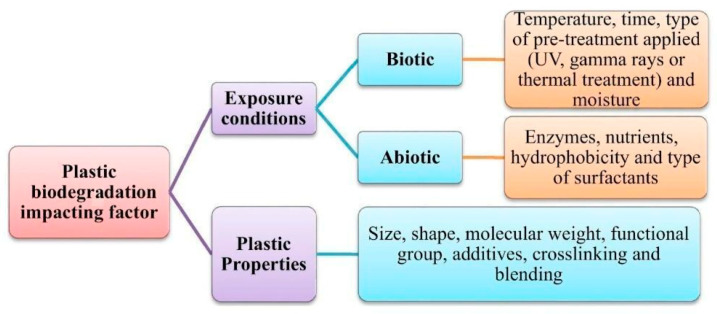
Biodegradation by enzymes on a typical thermoplastic (e.g., polypropylene): factors that influence the degree of decomposition/depolymerization (modified from [65], correcting a few spelling errors, such as in “biodegradation” and “nutrients”).

The definition of “inherently biodegradable” polymers is explained in Figure 5, while considering that any polymer material could be biodegradable in a designed environment allowing creatures to produce enzymes able to consume and ultimately mineralize them. This of course includes bio-based versions of the most widespread thermoplastics, such as polyethylene and polypropylene. A well-known example is the decomposition of polystyrene through biodegradation by means of mealworm (*Tenebrio molitor*) [66]; an earlier study derived the toxic effects (a proof of actual interaction) on it produced by naphthalene, which raised concern about all benzene derivatives [67]. When polymerizing styrene, these toxic effects disappeared, but an interaction was confirmed. Moving on to polymers of interest in the automotive industry, polypropylene can be decomposed by a number of bacteria with an adapted incubation process [68], though a number of factors can also influence this process, as reported in Figure 6.

Returning to Figure 5, the two sectors to the right include (top) materials that are more frequently referred to as “bioplastics”, being both bio-based and inherently biodegradable, and (bottom) petrochemical polymers that have been transformed to be more biodegradable. In itself, biodegradability is a detrimental factor for structural applications, yet, even without considering end of life, it can make materials more easily processable for some specific technologies, such as rapid prototyping. This is the case, for example, for poly(butylene adipate-co-terephthalate) (PBAT)/polyglycolic acid (PGA) blends [69]. Some petrochemical polymers have also been grafted using specific bio-based agents, such as succinic acid for poly(butylene) to obtain poly(butilene succinate) (PBS) in view of their application with lignocellulosic fibers [70]. The most popular biopolymer is definitely poly(lactic acid) (PLA). Beyond its widespread applications in packaging, commodity plastics, the biomedical sector, and 3-D printing [71], its introduction into the automotive sector is gradually increasing [72], leading to the potential for a car’s entire body to be produced through additive manufacturing [73]. This would also help overcome some typical polymer limitations, such as scarce wear resistance [74].

## 5. Biomass-Developed Materials in the Automotive Industry: The Case of Bumpers 

### 5.1. The Variable Success of Lignocellulosic Fibers in the Automotive Industry 

The introduction of lignocellulosic fibers into the automotive industry has been considered a long-standing problem because, on the one hand, they are widely recognized for improving the environmental profile of automobiles, while, on the other hand, they face a number of difficulties regarding practical applications. These include first and foremost the variability of supplies regarding quantity and quality [75], a commonly discussed drawback, and other disadvantages have also been discussed, such as ineffective interfacial bonding and barely controllable water and moisture absorption [76]. The latter depends on the cellulose content of the fiber, on which quite comprehensive data are offered, e.g., in [77], where significant variations by cultivar are also reported. As a matter of fact, the introduction of biopolymers in combination with lignocellulosic fibers has proven difficult, so in practice most natural fiber composites are still produced using epoxy matrices to ensure the reliability and vast diffusion of the latter [78]. The development of various bio-epoxies has altered the balance even further in favor of their application in this context, though caveats can be identified regarding the fact that most patented products only include a limited amount of bio-based content [79]. However, further reductions in environmental impact can be achieved due to the fact that bio-based precursors are often extracted from biomass, such as tannins, vanillin, or gallic acid [80].

It is also expected that the introduction of biomass into automotive components could possibly also result in economical benefits and hence cost reduction. However, the initial approach, based on some well-established crops, only proved effective in some local situations, such as in the case of Mexico with henequen, involving an articulated system of incentives to growers and investors [81]. More recently, an approach based on fibers as by-products or waste from larger economical systems singled out the automotive industry as a high-added-value sector, which drastically changed the situation [82]. It is highly probable that the two different methods for the production of automotive components—the use of specially developed lignocellulosic reinforcements and the upcycling of secondary raw materials from biomass—could coexist at some point, with a choice being made based on balancing economical considerations and performance.

Countless types of fibers have been trialed in the automotive sector, and the amount of attention dedicated to this field of study is clear from the number reviews that widely mention or completely focus on automotive applications, e.g., on flax [83], bamboo [84], hemp [85], sisal [86], jute [87], banana [88], pineapple [89], kenaf [90], and date palm [91]. Other explorative studies have been carried out on more niche fibers, such as curauà, using a polypropylene matrix, which are therefore specifically aimed at the automotive industry in view of the use of biopolypropylene from sugarcane ethanol [92]. It is worth noting that the economic system surrounding sugarcane offers enormous amounts of lignocellulosic waste, up to around 540 million metric tons per year, mainly in Mexico and Brazil yet gradually increasing worldwide, even in the warmer Mediterranean areas [93]. This is one of the most experimentally investigated biomasses, and it has also been proposed for introduction into the automotive industry as sugarcane bagasse, which has shown promising properties in terms of acoustic and thermal insulation. It has also shown good outcomes when coupled with other largely available types of waste, such as bamboo charcoal [94]. More specifically, the production of bagasse using a compression molding process has been proposed. It could be used in combination with palm sheath fibers in an epoxy matrix, with a total fiber content equal to 40 wt.% and a ratio of 40:60 between palm sheath and bagasse, with the aim of producing a composite dashboard [95]. As indicated above, the local availability of niche fibers is a continuously developing matter. Recent examples include the use of canola (*Brassica napus* L.) [96], which plays a large role in the industry as a rapeseed oil product, or cattail (*Typha* spp.), which has been suggested for use in devices aimed at improving energy storage in vehicles [97].

**Figure 7:**
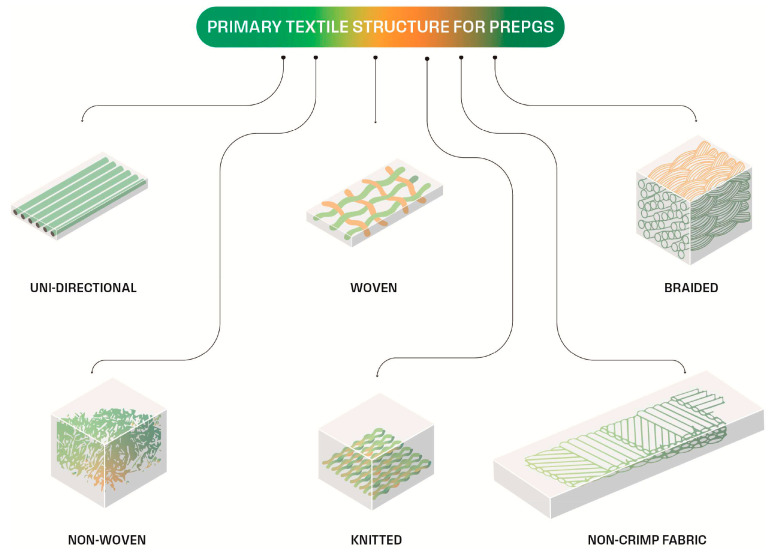
Textiles for pre-pregs (modified from [98]).

However, the practical application of lignocellulosic fibers arranged in continuous rows in structural automotive composites requires the development of textiles, such as those proposed in Figure 7, which can then be developed into pre-impregnated sheets (pre-pregs) to facilitate the molding process [98]. When only short fibers are available, different manufacturing techniques are possible, such as resin transfer molding (RTM) or injection molding, as previously discussed, which results in limits to the complexity of the structure.

Pre-pregs, including lignocellulosic fibers, have been produced using flax and have a range of applications, using, e.g., epoxy [99] or, more recently, poly(lactic acid) (PLA). PLA, on the one hand, has better thermal stability and fire retardancy to the extent that it is considered a thermosetting substance [100], as determined by its end-functionalized star-shaped structure and grafting. Another possibility is applying the pre-impregnation process to the filament, which is the method used for a “3D printed car” [101].

The development of geometrically sound and regular pre-pregs indicates the need for a considerable focus on the controllable parameters of the curing process, which can only be realized by reducing discontinuities in flax fibers and resolving the possible unevenness of their impregnation. In the specific case of automotive applications, the production of hybrid composites, including flax fiber pre-pregs layered alongside carbon fiber ones, indicates the large effect of the stacking sequence on falling-weight impact properties and the appearance of visible damage, with the external location of flax layers resulting in degraded material properties [102]. It has been proposed that the widely recognized issue of impregnation could be addressed by obtaining acrylic resin emulsion to interact with jute fibers, though this is only possible with a limited volume of reinforcement and large differences in tensile strength, depending on fiber orientation [103]. The effect of surface treatment is equally significant, as was revealed both with alkali and with laccase fiber modification on jute twine: the latter was particularly effective on composites for pipe winding, and it led to a nominal pressure of up to 2.88 MPa [104]. Chemical treatment could be discarded in favor of more sustainable processes, such as tannin/hexamine treatment, which allows for the production of pre-pregs from flax with up to 50% fibers using slow curing at a low temperature (130 °C for 35 min) with a moisture content of 20% on dry material [105].

The large number of fibers proposed does not necessarily correspond to their distinct applications in the structural body of a car: one of the few examples of their application to the wider concept of a “green car”, including solar panels, is the Eco-Elise [106]. On the other hand, even if a number of potential applications of natural fiber composites in the automotive industry are declared as offering an end-of-life scenario involving biodegradation with the ability to ensure safe and active disintegration by soil burial, studies on this specific aspect are still limited. One specific study involved reinforcing PLA with progressively increasing amounts of nettle fibers (up to 90 wt.%) to ensure weight loss after soil burial for 45 days: in this case, nettle showed a much larger weight loss than PLA and, generally, greater hydrolysis, whilst bare PLA only lost around 6% mass in the same period of time [107].

In this context, it is important to note that, despite the most common polymer for automotive applications being polypropylene, epoxy has been revealed as being more reliable when a “new” lignocellulosic fiber has to be introduced that has never or seldom been used before for composite reinforcement [108]. It has even been suggested that epoxy is particularly adaptable in terms of its compatibility with particulate waste blends, such as those extracted from *Moringa oleifera* waste pods with cellulosic short fibers [109]. Also, some specific characteristics of cellulosic particles, such as their improved wear resistance, have also been exploited by inserting up to 15 wt.% of sisal/paper pulp into an epoxy matrix, although this property is affected by the filler distribution [110]. A study on sisal inserted as a reinforcement for a bio-epoxy with a cardanol-based crosslinker and a total bio-content of 65% proved promising for potential shock-absorbing applications in the automotive industry [111].

### 5.2. The Case of Bumpers

The production, even if prospective, of a bumper, devised for a few decades, is considered a breakthrough for the widespread application of lignocellulosic fibers in this sector [112]. A limitation of this approach is based on the quality of the biomass used, but it is widely available. This results in studies with contradictory results, such as the modeling of polypropylene with empty palm fruit bunch (EPFB) fibers with no declared dimensional control [113]. However, comparison studies are being carried out in terms of multiparameter selection focused on car bumper use, including impact energy absorption properties. In [114], based on a polypropylene matrix, flax demonstrated globally higher properties with respect to kenaf and sisal, though the different dimensional controllability of the three fiber textile structures might have also played a role in this. The properties considered for comparison included density, mechanical properties (tensile strength, Young’s modulus, flexural strength and modulus, and impact strength), and some coefficients for production and end of life, including manufacturability, recyclability, sustainability, and disposal.

Following on from previous considerations, this section deals with attempts to produce car bumpers using materials that have been developed using biomass at some stage, either as the raw matter used to synthesize the biopolymer (typically thermoplastics) or, more frequently, that used to produce the reinforcement (lignocellulosic fibers), or even both. Given the involvement of biomass in bumper production at some stage, the possibility of hybridization needs to be considered. One example is the reinforcement comprising glass and lignocellulosic fibers lined up next to each other [115]. Hybridization with short glass fibers was also proposed with the use of up to 10 wt.% cellulose pulp fibers in order to elucidate the potential for injection molding, especially considering the use of a possible blend polymer between polypropylene and polyamide-6 (PA6) in the automotive industry [116]. This practice is particularly suitable for structures such as bumpers, where simplicity is often abandoned, as shown in the design in Figure 3, to instead try to achieve tailored and multi-material processing, especially as far as energy absorption and hence impact performance are concerned [117]. It is worth noting that many studies are numerical, which do not always involve the development of a prototype.

Due to obvious difficulties in terms of certification over crashworthiness, the first attempts to introduce biomass into car bumpers concerned hybrid glass/plant fiber composites. This was the case for glass/kenaf composites [118] (two layers of kenaf alternated with three layers of glass fabric, impregnated with an epoxy resin produced by compression molding under a pressure of 80 bar at 85 °C for 1 h), whose performance was compared with glass mat thermoplastics (GMT) (Table 2). The principal limitation concerned the impact properties, while tensile and flexural strength were improved; the reduced cost of the kenaf fiber allowed a woven glass fiber structure to be applied instead of the mat for the hybrid.

Another study indicated that kenaf composites could replace fiberglass sheet molding compounds (SMCs) for automotive applications, overperforming them in terms of tensile and flexural strength after the addition of 12% zinc oxide nanoparticles at the expense of slightly increased density [119]. It is also worth mentioning that, as reported in [120], SMCs have one of the lowest energy consumption values among all composite making techniques, around 3 MJ/kg, while the very effective and high-performance pre-preg procedure is at the other end of the spectrum, with 40 MJ/kg of energy consumption.

Other attempts to try to find the most suitable stacking sequence for bumpers have been carried out focusing on different fibers. In particular, a vehicle bumper was developed using a bamboo fiber-reinforced composite hybridized with jute fibers in an epoxy matrix [121]. The study concentrates on various stacking sequences, from five layers of jute (JJJJJ) to eight layers of bamboo (BBBBBBBB), through various hybrid combinations, namely JBJBJBJ, BJBJBJB, and BBJBBJBB, keeping constant the total amount of reinforcement to 30 wt.%. Tensile and flexural testing demonstrated that JBJBJBJ offered in both cases the highest properties. In particular, values of 47.4 MPa and 695 MPa were obtained for tensile strength and modulus, respectively, while flexural strength and modulus were equal to 80.2 MPa and of 9.065 GPa, respectively. This promising result was attributed to the lower extent of fiber pull-out detected. Further analysis on possible bumper applications was performed using a sisal–coir hybrid composite, with up to 40 wt.% fibers, though this did not provide fully conclusive results [122]. Another modeling contribution to the development of bumpers concerns applying the gradient distribution method to bamboo and cattail structures to limit lateral impact damage [123]. The model showed a reduction of 33% in crush deformation, alongside a 44% reduction in the total weight of a conventional thin-walled structure using the same materials.

**Figure 8:**
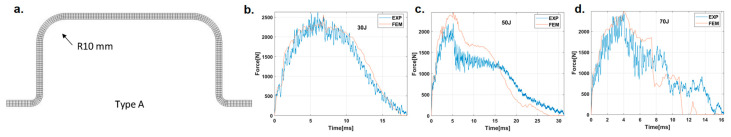
(**a**) Bumper beam design, falling weight impact hysteresis curves, and relevant simulation. (**b**) Impact at 30 J; (**c**) impact at 50 J; (**d**) impact at 70 J (from [127]).

From these preliminary results, it seems that the focus is understandably on impact properties, specifically the bidimensional (falling weight) impact mode [124]. Here, the hysteresis curves offer valuable information not only on the percent of energy absorbed elastically (linear stiffness) [125], plastically, and during a possible rebound, but also on the vibration characteristics of the beam [126]. In [127], a study on flax–PLA bumpers indicated non-negligible differences between the finite element simulation and the hysteresis curves of the falling weight impact at different energies, as shown in Figure 8. This can be explained by the irregular geometry of the reinforcement, including internal lumens, which induce complex vibration modes. Despite these limitations, flax has been reported as being one of the most suitable options for introduction into bumpers for its high tensile strength and ability to introduce large amounts of fibers into textile structures. In [128], a composite bumper with 43 wt.% flax fibers is reported to have a 205 MPa tensile strength and 158 MPa flexural strength, while absorbing without penetration 45 J of energy. This is overwhelmingly superior to the competitor fiber proposed, sisal, which, on the other hand [129], proved extremely suitable for its ability to perform well in different fiber orientations due to its mold compressibility for less loaded structures, such as automotive fenders (possibly an inferior and semi-structural part of the bumper). Notwithstanding these limitations, sisal, in combination with other fibers (e.g., curauà) and in regional contexts close to production fields, remains a candidate for the prospective manufacturing of automotive bumpers [130]. Other attempts have been made recently using other hybrid structures, such as a jute/oil palm fiber structure [131], coupled to a textile fiber with typical waste biomass. This was carried out to tailor the composite’s performance in terms of its falling weight impact with the respect to the amounts of the two reinforcements.

Other data are available for bumper beams fabricated using hemp fibers [132]: in particular, a study on stacking sequences for hybrid hemp/glass composites was performed, and through optimization, they were able to absorb up to 90% of the energy absorbed by a glass fiber composite; meanwhile, it was suggested that a ±45° fiber orientation was more effective than a 0°/90° one [133]. Hemp fiber composites have traditionally garnered interest in the automotive industry for the high tenacity and shear resistance of their textile products, even when processed with larger flax looms [134]. More recently, the potential of hemp fibers has been further highlighted, which is again of interest for the automotive industry, especially in terms of flame retardancy. They can also be used in combination with bio-based resins, such as bio-epoxy [135].

**Figure 9:**
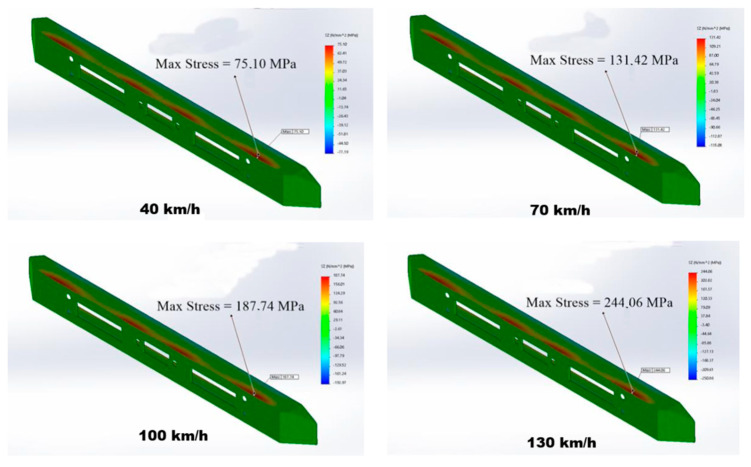
Simulation of a composite bumper with pineapple leaf fiber (PALF) reinforcement [138].

Another reinforcement that has been investigated for the purpose of bumper production is pineapple leaf fibers (PALFs), which, due to their higher geometrical regularity than other plant fibers, can be introduced into the composite in greater amounts, such as 30% or more, even in the absence of a textile structure, with advantages in terms of impact resistance [136]. The main limitation of PALFs is their large amount of cellulose, second only to cotton amongst the most commonly used fibers, which results in the need for thorough control of water absorption and the application of protective treatments [137]. To better define the potential application of PALF composite bumpers in a specific context, in [138], the Isuzu Elf NKR55 Microbus setting was selected. In this way, the critical stresses and strains of the designed bumper were measured, and it was indicated, by evaluating the elastic limit of the composite, that a PALF composite bumper could withstand penetration in a car for an impact at a velocity of up to 70 km/h. Although precise data on compliance with EN-R42 regulations were not obtained, regarding low-speed impacts and the gradual process of composite damage, this result is sufficient to demonstrate the adaptability of the bumper to most situations, namely urban traffic. The simulation carried out is reported in Figure 9.

It is noteworthy that, in addition to the previously mentioned fibers, other fibers have also been proposed for use in bumpers. One of the first lignocellulosic fibers investigated in the literature for use in composites was jute [139] due to its distinct advantages, namely producing flexibility in textile products such as carpets, rugs, sacks, ropes, etc. Jute is also of particular interest for forming hybrids with various fibers, not only limited to glass, with various stacking sequences [140]. This could extend to the majority of lignocellulosic fibers [141]. It is no surprise that a rear bumper made from a jute fabric and orthophthalic polyester composite has been proposed and prototyped, though it was not able to withstand an impact at 4 km/h, unless its thickness, initially set at 5 mm, was considerably increased [142].

Recently, the number of lignocellulosic fibers used in composites for bumper manufacturing has been increasing. A study on plantain (*Musa paradisiaca*), often associated with banana in composites [143], reported the introduction of up to 40 vol.% of fibers into the composite. The higher the tenor of the fibers, the more limited their advantages were. In particular, an unexceptional impact strength of 13.83 kJ/m^2^ was reported for the most fiber-charged composite [144]. It is also recognized that the fact that it belongs to a large, food-based production system promotes its potential use in the automotive industry for some fibers. A comparison between composites with 30% short okra [145] and banana fibers indicated that, while the highest properties were obtained for the former at a 10 mm length, for the latter, fibers of up to 50 mm did improve the composite’s performance [146]. This indicates, on the one hand, the larger effect of defects in okra than in banana fibers, yet on the other hand it reveals that these fibers could both be considered candidates for potential structural use in composites for automotive applications.

Another possible approach is based on the availability of a large and articulated productive system to select potential fibers for bumpers. In this sense, bamboo, though not the most adaptable fiber in composites due to its complex modes of fracture [147], would appear a natural choice. It offers long fibers with a reasonably constant diameter and a relatively high impact strength (up to 46 kJ/m^2^ for an epoxy bamboo hybrid with 40 wt.% fiber content, with fibers oriented at 0°, but going down to only 14 kJ/m^2^ for fibers oriented at 90°) [148]. The variability of impact strength with fiber orientation obviously represents a significant issue for the fabrication of complex geometries, as in the case of bumpers.

## 6. Future Perspectives and Conclusions

The automotive sector is becoming and will increasingly be in the future more likely to utilize not just recyclable materials but also polymers and polymer composites sourced from biological origins, including plant- and biomass-based materials to facilitate decarbonization and reduce resource depletion [149]. In this sense, it is fair to say that the drive towards using bio-based materials in the automotive industry is steadily growing. For instance, Ford showed interest in employing soy-based polyurethane foam for seats two decades ago [150], whereas LANXESS supported the utilization of bio-based poly(butylene terephthalate) (PBT) sources from dextrose [151]. The use of bio-polymers in the automobile industry is promising since they are renewable and minimize petroleum consumption. Recently, legislation in Asia, Europe, and the United States has encouraged the use of naturally produced biomaterial or recycled materials in the production of automobile parts [152]. The use of PLA is also increasing due to the gradual rise in 3-D printing methods, such as fused filament fabrication (FFF), beyond prototyping, e.g., in the production of spares for various industries, and therefore it is also increasing in the automotive industry, e.g., Volvo’s use of foamed PLA [153]. A major potential competitor of PLA, bacterial poly(hydroxyalkanoate) (PHA), which is heavily dependent on biomass for feedstock, is also being considered for use in automobile parts, with the addition of thermal stabilizers, such as nanoclay, required to compensate for the higher heat deflection of PLA/PHA [154]. Though quite slowly, considering the examples traced back to two decades ago, biopolymers are being increasingly used in the production of various components of vehicles, even for bumpers. For example, the rear bumper stay of Mazda’s CX 3 and Mazda 3 models utilizes BASF’s “Ultramid Balance,” a partially castor oil-based polyamide (PA6/PA6.6) material, competing against petrochemical polyamides in safeguarding the vehicle’s underbody against road salt and exhaust heat [155]. In general terms, though, this introduction of biopolymers, as suggested above, is much slower than expected: drawbacks and hindrances are becoming increasingly more apparent. In itself, the combination biopolymer + biomass would appear an ideal substitute for traditional composites; a number of difficulties arose with time nonetheless. Examples are the difficulty of adapting hierarchized biological structures to the dimensional tolerance limits of advanced and serial industrial structures, such as car bumpers, and the limited knowledge of materials engineers of certain factors, e.g., the relationship between the cultivation process and the final properties, even for fibers that have been widely proposed for automotive use, such as flax and hemp [156].

Recently, a concealed yet steady paradigm shift has gradually been taking place. The overwhelming amount of biomass available for use in materials, as opposed to bio-fuel and bio-energy, suggests, on the one hand, a greater focus on its use as a secondary raw material; however, the number of species available has increased. While this process reduces costs, it also reduces the practical applicability of tools such as lifecycle analysis (LCA) by hindering the traceability, including initial processing and transportation, of agricultural waste biomass [157]. However, the large-scale use of biomass would need to involve the structural application of lignocellulosic fiber composites in automobile production. A large number of attempts, proposals, numerical studies, and prototypes focus on a vehicle’s interior components and parts, with a particular emphasis on the development of dashboards [158]. In this case, the non-criticality of its design, intended only as a cover, allows a larger tolerance for variabilities, and the “woody” aspect offered by some fibers might be even an incentive for the application of this method. In this sense, some of the largest automotive companies have even showed some interest in non-structural applications; for example, Ford’s Flex incorporated wheat straw-reinforced polypropylene into its design. The interior storage bins utilized approximately 20% wheat straw, resulting in components that were 10% lighter compared to those made with talc-filled polypropylene, notwithstanding the more limited (yet not strictly required) resistance to abrasion [159].

Despite the increasing amount of biomass available for production, essentially at no cost, especially when considered as a secondary raw material or residue, its introduction into structural parts of vehicles has still been quite slow. In this sense, the development of a bumper demonstrates the effectiveness of the introduction of agricultural biomass, not just some specific textile fibers, into the automotive industry. However, the literature on lignocellulosic fibers for use in composites provides contrasting evidence. Some studies concentrate on the idea of improving the regularity of some specific fibers, which demonstrates more success in composites. This is the case, e.g., for hemp, especially via different treatments to optimize performance [160], or for flax via the production of specific impregnated thermoset tapes adapted for use in the automotive industry [161]. There might also be scope for the application of other, less well-known fibers [162], especially in view local biomass supply and fibers being by-products of large systems, such as the those based on banana [163].

To conclude, designing bumpers including biomass can be a useful exercise, independently from their immediate application [164]. It is important to clarify the ideal stacking sequence and the effect of fiber orientation while also modifying the geometry with respect to traditional GMT/polypropylene bumpers, even if this might not be totally compatible, at least at first, with crashworthiness requirements. It has also been suggested that further possibilities could be offered by the development of additive manufacturing in the automobile industry, using biomass-filled PLA filaments for production [165]. Prototyping and simulation are highly necessary with regard to impact performance, and it is worth considering that a number of different biopolymers might become available over time for the development of this crucial component of vehicles.

## Figures and Tables

**Table 1:** Some considerations from early reviews on the application of lignocellulosic fibers in the automotive industry.

Highlighted Potential/Criticalities	Ref.
Applying maleic anhydride-grafted polyolefins is necessary to introduce natural fiber thermoplastics into automotive interior (possibly exterior) components.	[20]
Chemical (lignin, cellulose, etc.) and physical data for key lignocellulosic fibers are strongly required.	[21]
Jute, sisal, banana, flax, hemp, coir, and kenaf have each been considered, along with their corresponding potential reduction in fuel consumption and thermal and acoustical insulation capabilities.	[22]
Bioplastics are generally mentioned. However, no indication of lignocellulosic fibers is given. This review may be useful for assessing the range of potential market penetration	[23]
An innovative perspective is reported on the selection of lignocellulosic fibers: the selection starts from the characteristics of the crops involved, including high-production ones, such as corn, sugar beets, castor beans, and switchgrass, and perennial ones, e.g., cassava, sugar cane, and general cellulose sources.	[24]
The potential use of nanocellulose and relevant extraction methods to confer better properties to lignocellulosic fiber composites and possibly facilitate the use of biopolymers are suggested.	[25]

**Table 2:** Comparison of properties between bumpers made from various materials.

Properties	Glass/Kenaf [118]	GMT [118]	Flax [114]	Sisal [114]
Tensile strength (MPa)	153	69	67	55
Tensile modulus (GPa)	7.5	5.2	6.7	4.8
Flexural strength (MPa)	225	150	63	52
Flexural modulus (GPa)	12	4.8	6.3	4.5
Impact strength (kJ/m^2^)	30	48	44	28

## Data Availability

In this study, no new data were created.

## Abbreviations

The following abbreviations are used in this manuscript:

EPDM	Ethylene–propylene diene monomer
FFF	Fused filament fabrication
GMT	Glass mat thermoplastic
LCA	Lifecycle analysis
PA6	Polyamide 6
PALF	Pineapple leaf fiber
PBT	Polybutylene terephthalate
PBTA	Poly(butylene adipate-co-terephthalate)
PGA	Polyglycolic acid
PLA	Polylactic acid
POE	Polyolefin elastomer
